# Research Progress on Intracranial Lymphatic Circulation and Its Involvement in Disorders

**DOI:** 10.3389/fneur.2022.865714

**Published:** 2022-03-14

**Authors:** Fan Chen, Xuan Xie, Liang Wang

**Affiliations:** Department of Neurosurgery, Tangdu Hospital of Fourth Military Medical University, Xi'an, China

**Keywords:** lymphatic circulation, central nervous system, tumor, perivascular space, meningeal lymphatic vessels

## Abstract

The lymphatic system is an important part of the circulatory system, as an auxiliary system of the vein, which has the functions of immune defense, maintaining the stability of the internal environment, and regulating the pressure of the tissue. It has long been thought that there are no typical lymphatic vessels consisting of endothelial cells in the central nervous system (CNS). In recent years, studies have confirmed the presence of lymphatic vessels lined with endothelial cells in the meninges. The periventricular meninges of the CNS host different populations of immune cells that affect the immune response associated with the CNS, and the continuous drainage of interstitial and cerebrospinal fluid produced in the CNS also proceeds mainly by the lymphatic system. This fluid process mobilizes to a large extent the transfer of antigens produced by the CNS to the meningeal immune cells and subsequently to the peripheral immune system through the lymphatic network, with clinically important implications for infectious diseases, autoimmunity, and tumor immunology. In our review, we discussed recent research advances in intracranial lymphatic circulation and the pathogenesis of its associated diseases, especially the discovery of meningeal lymphatic vessels, which has led to new therapeutic targets for the treatment of diseases associated with the intracranial lymphatic system.

## Introduction

The lymphatic system is the second vascular system in the body after the blood circulation, consisting of lymphatic vessels, lymphatic tissues, and lymphatic organs, with functions such as regulation of tissue pressure, immune prevention, absorption of fat from the small intestine, and removing substances produced by cell death or metabolism (1, 2). The lymphatic system plays an essential role in inflammatory diseases, malignant tumor metastasis, lymphedema, and other diseases (1). Unlike the peripheral circulation, it has long been assumed that the central nervous system (CNS) does not have anatomical lymphatic vessels lined with endothelial cells, nor does it have a lymphatic circulation similar to that of the periphery, with a similar function in the brain manifested by the return of metabolic waste to the blood *via* the arachnoid granules. Louveau et al. (3) identified meningeal lymphatic vessels located in the inner layer of the dural sinus within the brain by whole fixation and immunofluorescence staining of the mice meninges. Aspelund et al. (4) found lymphatic vessels lined with endothelial cells in the dura mater by immunofluorescence staining of the skull, dura mater, arachnoid, and soft meninges of wild juvenile mice. Moreover, it was confirmed that the structure of the meningeal lymphatic vessels may be related to the circulation of immune cells and the absorption of cerebrospinal fluid (CSF) (3–6). The development of the lymphatic system involves many endothelial markers, such as prospero-related homeobox-1 (PROX1) (7), SRY-related HMG-box transcription factor 18 (SOX18) (2), and nuclear receptor COUP-TFII (NR2F2) (8) related to lymphatic differentiation; podoplanin (PDPN) and lymphatic endothelial hyaluronic acid receptor 1 (LYVE-1) related to lymphangiogenesis (9, 10); and vascular endothelial growth factor 3 (VEGFR-3) and VEGF-C related to lymphatic sac formation (11–13). Studies have demonstrated that lymphangiogenesis is closely related to tumor progression and plays an important role in tumor infiltration and metastasis, and lymphatic vessels can be considered as sites of future tumor metastasis in tumor individuals (14, 15). For example, VEGF-C and VEGF-D are closely associated with tumor-associated lymphatic vessel formation, which can lead to tumor metastasis to the sentinel lymph nodes (15, 16).

Meningeal lymphatic vessels have been shown to express the full molecular signature of lymphatic endothelial cells, can carry immune cells and fluid in the cerebrospinal fluid, communicate with deep cervical peripheral lymph nodes (17). However, the detailed network structure and characteristics of the meningeal lymphatic network are still not particularly well understood, and the relationship between meningeal lymphatic vessels and the development of several pathological conditions, especially the development of tumors, is unknown. We analyzed the development of the lymphatic circulation in the CNS and the relationship between meningeal lymphatics and CNS diseases. A better understanding of meningeal lymphatics may provide new directions for the treatment of immune-related CNS diseases and CNS tumors.

## Lymphatic Vessels in the Cns

### Historical Background

In the past, it was thought that although there were no lymphatic vessels in the CNS, there was lymphatic drainage, and lymphatic drainage was mainly through the perivascular space (18). This space was first found by the German pathologist R Virchow in 1851 and the French biologist C P Robin in 1859, and was later named Virchow–Robin space (VRS). In 1989, Wang et al. (19) demonstrated the existence of a prelymphatic system in the vascular epithelium of the internal carotid artery and its branches, which can drain intracranial lymphatic fluid into the cervical lymphatics, and confirmed that the prelymphatic system of the cerebellar lymphatics is located in the vascular epithelium of the vertebrobasilar artery system. In 1996, Li et al. (20) identified meningeal stomata with electron microscopic scans, distributed among meningeal mesothelial cells, and confirmed that meningeal stomata may be part of the prelymphatic capillary system of the brain. In 2004, Johnston et al. (21) confirmed an association between CSF and nasal lymphatic vessels in humans and other mammals. In 2005, Gao et al. (22) found that when the tracer was injected into the cerebral cortex of rabbits, the tracer could appear in the deep neck lymph nodes. In addition, when the tracer was injected into the deep neck lymph nodes, it could also appear in the brain tissue and CSF. Marín-Padilla et al. (23) reported that the intracerebral prelymphatic system originates from the VRS, which is used as a prelymphatic drainage system for the draining of substances that cannot be transported into the catabolized or blood intracellularly, is closely associated with the cortical penetration of small arteries and veins, and is an inefficient primitive lymphatic system lacking the anatomical and physiological components found in the peripheral lymphatics. In 2012, Begley (24) and Iliff et al. (25) proposed the glial-lymphatic pathway hypothesis and confirmed the existence of the glial-lymphatic pathway by injecting a fluorescent tracer into the lateral ventricles of mice and confirmed that its circulatory power is derived from arterial pulsation and intracranial pressure. In 2013, Xie et al. (26) found that the glial lymphatic system can serve as a key regulator of brain metabolic waste removal during sleep. In terms of lymphatic fluid formation, the VRS is similar to the peripheral lymphatic system in that interstitial fluid (ISF) enters the VRS to form intracerebral lymphatic fluid. Generally, CSF drains to lymph nodes through the lymphatic vessels around the intracranial nerve roots or is absorbed from the arachnoid granules into the circulation, while the ISF forms intracerebral lymphatic fluid drains to the cervical lymph nodes along the outer wall of the vessels (27). The perivascular cells present in the VRS and perineural space are bone marrow-derived cells with phagocytic and antigen-presenting abilities that can be continuously renewed by peripheral mononuclear macrophages to clear foreign bodies from the VRS and perineural space (28). As the most important part of the classical CNS lymphatic circulation, the VRS also contains dendritic cells derived from hematopoietic stem cells, which have strong antigen-presenting ability. When activated, leukocytes can adhere to the endothelium through the ligand and enter the VRS and brain parenchyma through the endothelium to perform immunosurveillance functions (29–31). In fact, as early as 1787, Mascagni proposed the possible existence of lymphatic vessels in the human dura mater in his “Vasorum lymphaticorum corporis humani historia et ichonographia”, and Lecco and Li et al. mentioned the related concept in 1953 and 1996, respectively. However, it was not confirmed in experiments due to the limited technical conditions at that time (20).

### Meningeal Lymphatic Vessels

**Figure 1:**
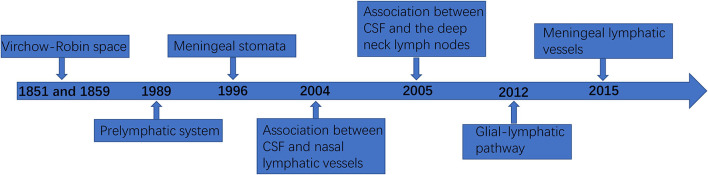
The timeline flowchart of the development of lymphatic circulation in the central nervous system (CNS).

Unlike the classical CNS lymphatic circulation, the discovery of meningeal lymphatic vessels further confirmed that the CNS is not an immune exempt organ, and has a peripheral-like immune function (4). The presence of a functioning lymphatic system in the extensive dura mater has been successively confirmed using two different studies in which the meningeal space and brain parenchyma drained macromolecules, CSF, and immune cells (4, 32). The meningeal lymphatic vessels are functional lymphatic vessels located in the inner layer of the dural sinus and structurally parallel to it, through which immune cells and fluid components of the CSF can be transported and connected to the deep cervical lymph nodes (3). The meningeal lymphatic network travels above the olfactory bulb and eventually converges into lymphatic vessels that run parallel to the dural sinuses (3, 4). Figure 1 shows the timeline flow chart of the development of lymphatic circulation in the CNS.

**Figure 2:**
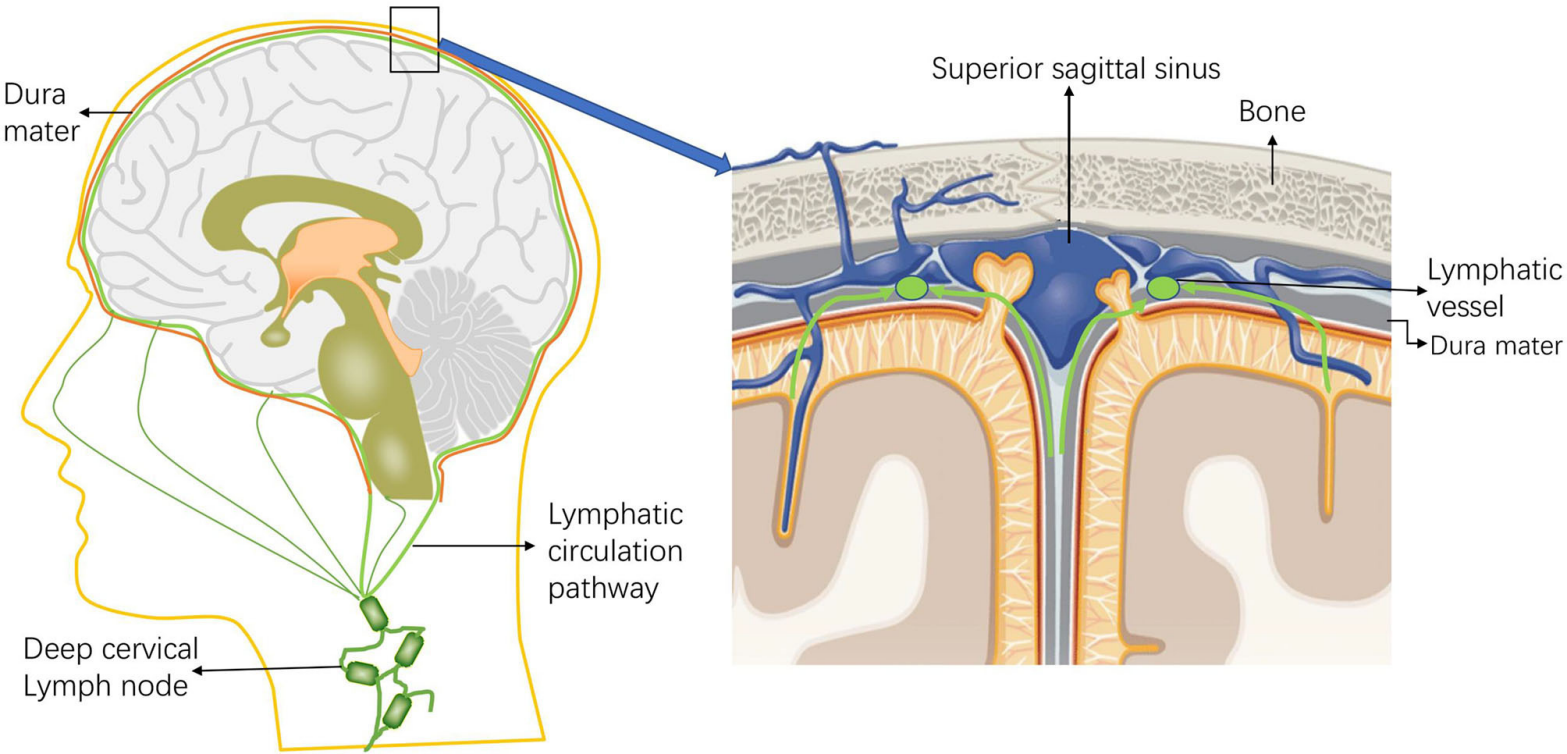
Schematic diagram of the intracranial lymphatic circulation and the location of the meningeal lymphatic vessels. Through the lymphatic system, the meningeal lymphatics absorb cerebrospinal fluid (CSF) from the adjacent subarachnoid space and cerebral ISF, and the meningeal lymphatics can deliver CSF to the deep cervical lymph nodes through the foramina at the base of the skull.

Interestingly, no connection between the meningeal lymphatics and the veins has been observed in mammals (2). New drainage pathways of CSF along the nasolacrimal duct into the lymphatic plexus of the esophagus and trachea were later also identified in animal experiments, and lymphatic vessels were found in the dura mater and in the dural septa that enter deep into the brain (33). Visanji et al. (34) found the presence of lymphatic vessels in the dura at the level of the superior sagittal sinus of the human brain by autopsy. In addition, immunohistochemistry of the lymphatic vessel endothelial cell marker podoplanin showed the widespread presence of multiple structures and the distribution of this unique lumen in the whole superior sagittal sinus (Figure 2).

The process of meningeal lymphatic vasculature development was well documented in animal experiments and was relatively late compared to other tissues (2). Published findings showed that in mice, essentially all intracranial and spinal lymphatic vasculature develops during the first month of life, starting at the base of the skull through the expansion of sprouts and the fusion of clusters of lymphatic endothelial cells located next to blood vessels and nerves (35–37). VEGF-C production in vascular smooth muscle cells, the pituitary and pineal glands is considered essential for lymphatic vessel development (35, 36). Application of VEGF-C/R3 gene targeting clinically approved VEGFR tyrosine kinase inhibitors or VEGF-C/D traps to inhibit VEGF-C-R3 signaling leads to developmental arrest of the meningeal lymphatics in pups and progressive regression of the meningeal lymphatics in adult mice (35). Intracranial delivery of exogenous VEGF-C *via* a viral vector induces rapid growth of meningeal lymphatic vessels in pup mice and further growth in adult mice (35).

*in-vivo* experiments have demonstrated that macromolecular substances in the brain, such as amyloid (I), can enter the periphery through the perineural space and meningeal lymphatic vessels and be metabolized in the periphery. Studies of mechanisms involved suggest that their transport is influenced by the blood–brain barrier, astrocyte aquaporin-4 (AQP4), and apolipoprotein E (38, 39).

Iliff's *in-vivo* two-photon imaging with small fluorescent tracers revealed that CSF enters the parenchyma along the paravascular spaces surrounding the penetrating arteries and that cerebral interstitial fluid is cleared along the paravascular drainage pathway (25). Animals lacking AQP4 in astrocytes exhibited slowed CSF inflow through this system and ~70% reduced clearance of interstitial solutes, suggesting that the large volume of fluid flow between these anatomical inflow and outflow pathways is supported by water transport from astrocytes, on which the concept of a glymphatic system was developed (25). Iliff and Nedergaard (40) previously found an ~65% decrease in Aβ clearance in mice by establishing an AQP4 knockout mouse model. Xia et al. (41) found that the intracranial lymphatic circulation pathway in mice was damaged under stress, and endogenous and exogenous Aβ42 accumulated in the brain of mice.

It has also been noted that the meningeal lymphatic vessels represent a system of vasculature that removes cerebrospinal and interstitial fluid to the cervical lymph nodes. Previous studies, on the other hand, have focused on the dorsal meningeal lymphatic vessels, which are located within the dural folds along with the superior sagittal and transverse sinuses. Since the dorsal meningeal lymphatic vessels do not absorb and drain CSF tracers, meningeal lymphatic vessels in other parts of the CNS remain unknown, such as the basal or lateral meningeal lymphatic vessels of the skull. Ahn et al. (42) demonstrated that the basal meningeal lymphatic vessels are more amenable to uptake and clearance CSF than the dorsal meningeal lymphatic vessels by dissection of the skull base of mice. They further showed that the basal meningeal lymphatic vessels are the main pathway of CSF macromolecule metabolism by functional assessment of meningeal lymphatic vessels by contrast-enhanced MRI and fluorescence imaging of the CSF.

### Immune Cells in the Brain Lymphatics

A large number of immune cells, namely, MHC II + cells, T lymphocytes, dendritic cells, and B lymphocytes, can be detected in the newly identified meningeal lymphatic vessels (43, 44). Louveau et al. demonstrated that meningeal lymphatic vessels are an important drainage pathway for CNS/CSF-derived soluble molecules and immune cells, and that in mice with multiple sclerosis, reduced lymphatic drainage under neuroinflammatory conditions led to a reduction in the acquisition of encephalitic properties by antigen-specific T cells, resulting in amelioration of clinical symptoms in mice with multiple sclerosis (32). Under homeostatic conditions, both mouse and human brain parenchyma lack lymphocytes, and sporadic lymphocytes are present in the leptomeninges, whereas CSF contains small amounts of CD4+ T lymphocytes, antigen-presenting cells (APCs), or monocytes (45). The absorption of CSF from the subarachnoid space and interstitial fluid by the meningeal lymphatics through the lymphatic system and the subsequent transport of the fluid to the deep cervical lymph nodes also suggest that the meningeal lymphatics are involved in the homeostatic transport of meningeal immune cells.

## Lymphatic Circulation of the Cns and its Related Diseases

### Lymphatic Circulation and Brain Swelling

**Figure 3:**
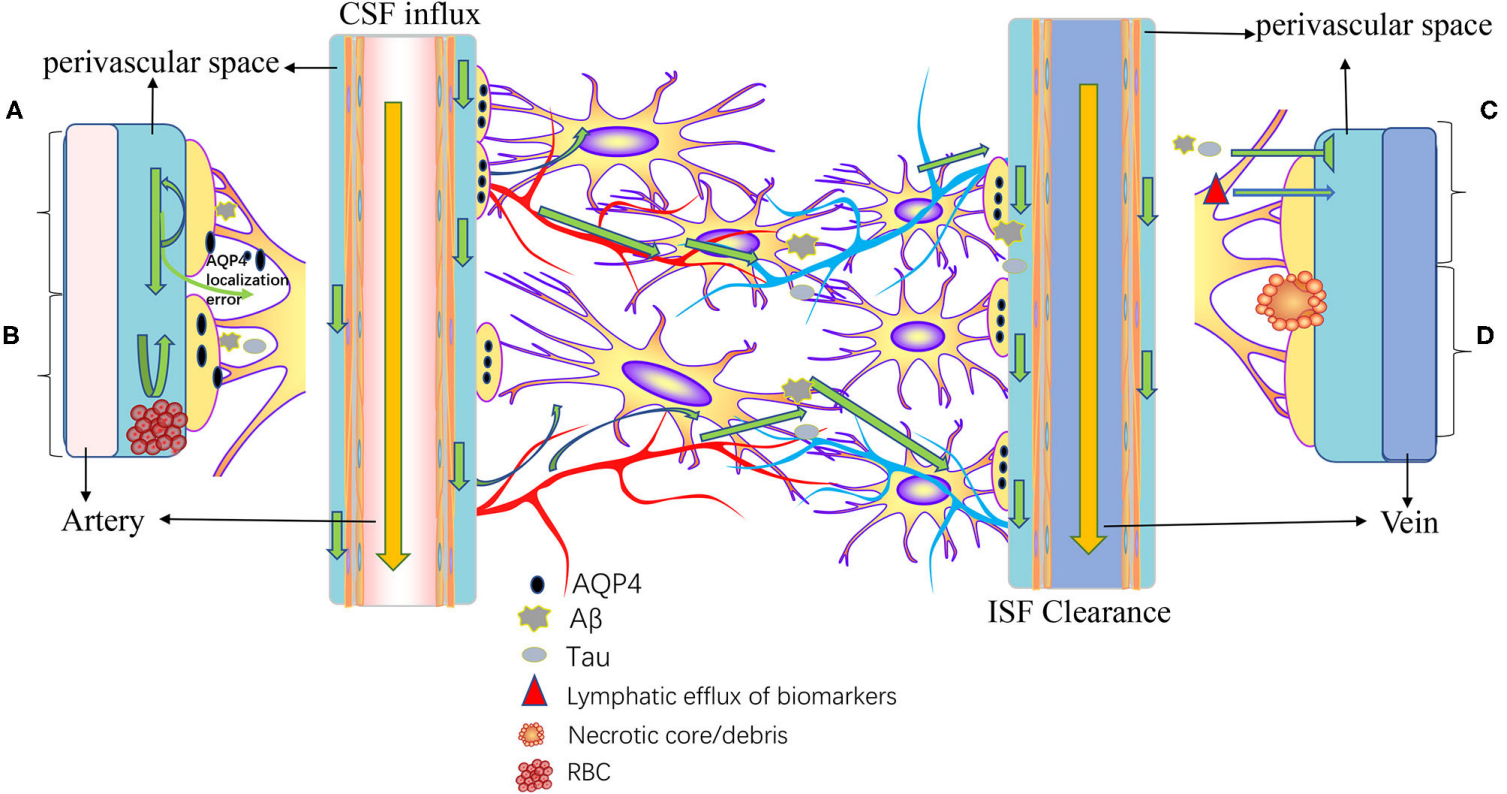
Pathogenesis of diseases related to intracranial lymphatic circulation. A, Neurodegenerative diseases; B, cerebral hemorrhage; C, traumatic brain injury; and D, stroke and small vessel diseases. In neurodegenerative diseases, Aβ causes mislocalization of aquaporin-4 (AQP4) and impaired CSF influx and reduced CSF clearance. In intracranial hemorrhage, infiltration of blood components into the perivascular space, particularly fibrin/fibrinogen deposition, occludes the perivascular space, resulting in decreased CSF inflow. Traumatic brain injury results in decreased lymphatic clearance and biomarkers of injured parenchyma can be transported to the cervical lymphatic system *via* intracranial lymphatic pathways. In ischemic stroke and small vessel disease, a necrotic core forms within the brain parenchyma, and the contents of the necrotic core leak into the perivascular space through permeable glial scarring. Cerebral microinfarction can lead to transient decreased lymphatic inflow and prolonged retention of solutes within the infarcted core.

The meningeal lymphatics can absorb CSF from the associated brain interstitial fluid and subarachnoid space through the lymphatic network, then deliver CSF to the deep cervical lymph nodes through holes in the skull base (3). CSF circulation through the meningeal lymphatics is associated with several CNS-related disorders, such as cerebral edema following trauma or cerebral hemorrhage, Parkinson's disease (PD), and Alzheimer's disease (AD) (43, 46, 47). Immune surveillance of the meningeal lymphatics is associated with CNS demyelinating diseases, such as multiple sclerosis (MS) (2, 48). In addition to this, meningeal lymphatic vessels have been reported to be associated with the development, spread, and metastasis of CNS tumors (49, 50) (Figure 3). Table 1 summarized the diseases related to the lymphatic circulation of the CNS and their mechanisms. Table 2 summarized the signals and molecules that induction of lymphangiogenesis. Brain infarction, hemorrhage, trauma, and tumors usually cause brain swelling and hydrocephalus (51, 52). Brain swelling and hydrocephalus from the above causes can increase intracranial pressure, leading to serious complications (2, 35). Previous studies found dysfunction of lymphatic pathways and impaired interstitial Aβ clearance in mouse models of mild and severe traumatic brain injury (53), mouse models of diffuse microinfarction (54), and the brains of aging mice (55). Iliff et al. (55) studied a mouse model of moderate to severe trauma by injecting a tracer into the perivascular cortex of mice after brain trauma, compared it to the ipsilateral trauma area, detected a significant reduction in tracer migration. This study confirmed that the activity of the lymphatic system in the posttraumatic mouse brain was reduced and that this phenomenon persisted 28 days after the injury, suggesting that there may also be an association between the impaired activity of the lymphatic system after injury and delayed brain swelling (55). The increase in meningeal lymphatics can reduce intracranial pressure by increasing CSF drainage (2), which may lead to new strategies for the treatment of brain swelling.

**Table 1:** Lymphatic circulation of the central nervous system (CNS) and its related diseases.

**Disease**	**Mechanism**
Brain swelling	Impaired activity of the lymphatic system
Neurodegenerative diseases	Lymphatic clearance dysfunction (clearance of Aβ and α-syn)
Neuroinflammatory disorders	CD4+ T-mediated inflammatory response
Small vessel diseases of CNS	Abnormal lymphatic drainage, Lymphatic clearance dysfunction (deposition of abnormal proteins)
Stroke	Lymphatic drainage and clearance dysfunction
Glaucoma	Lymphatic drainage and clearance dysfunction
Tumor immunology	Immune cells infiltration

**Table 2:** The signals and molecules that causes induction of lymphangiogenesis.

**Tumor and Inflammatory**	
VEGF signaling	VEGF-A, VEGF-C, Neuropilin-2
Alternative angiogenic signaling	PDGF, FGF, EGF, HGF, IGF-1/2, Ang1/Tie2, Adrenomedullin, Sphingosine-1-phosphate
Inflammatory molecules	TNF-α, Lymphotoxin-α, Toll-like receptor, NF-κB, Erythropoietin, COX-2, prostaglandin E2

*PDGF, platelet-derived growth factor; FGF, fibroblast growth factors; HGF, hepatocyte growth factor; IGF, insulin-like growth factor; TIE, Tie-receptor tyrosine kinase*.

### Lymphatic Circulation and Neurodegenerative Diseases

In recent years, *in-vivo* experimental studies have confirmed that meningeal lymphatic dysfunction plays an important role in the development of neurodegenerative diseases, with AD and PD being the most common and well-studied neurodegenerative diseases (56).

In AD, extracellular Aβ aggregation and intracellular tau neurofibrillary tangles are the disease's two major and representative neuropathological characteristics, which are the basis for AD classification (57). Shahnur et al. (58) found that Aβ deposition in the cerebral cortex was associated with dysfunction of the meningeal lymphatic system by injecting fluorescently labeled Aβ into the brain tissue of AD model mice. Other scholars have confirmed the view of impaired interstitial fluid dynamics in AD through animal experiments (5, 25, 59). Iliff et al. (25) evaluated brain Aβ excretion in healthy and AQP4 knockout mice before the discovery of meningeal lymphatic vessels. It found that the fluorescently labeled Aβ was transported along the perivascular pathway, and deletion of the AQP4 gene inhibited the clearance of Aβ, suggesting that the lymphatic pathway may be involved in the clearance of Aβ in the CNS. After ligation of deep cervical lymph nodes in AD model mice, the AD-like phenotype was significantly exacerbated, including more severe brain Aβ accumulation, impaired AQP4 polarization, synaptic protein loss, cognitive-behavioral deficits, and neuroinflammation (59). In a study on CSF clearance detection in patients with AD, 11C-PiB PET protein, which can image amyloid, was utilized and significant differences were found in patients with AD and healthy controls, suggesting that defective CSF clearance in AD is associated with Aβ deposits (60). The above results suggest that lymphatic clearance dysfunction is one of the key factors in the progression of AD, and repairing meningeal lymphatic drainage function can be a target for the treatment of AD.

In PD, previous studies have shown that meningeal lymphatic endothelial barrier dysfunction was associated with pathological α-synuclein (α-syn) meningeal macrophage-induced meningeal inflammation, which may ultimately lead to abnormal meningeal lymphatic vessel function (61–63). Ding et al. (56) reported that intrathoracic injection of α-syn increased the severity of PD-like pathology in mice after blocking meningeal lymphatic drainage. Zou et al. (64) blocked meningeal lymphatic drainage in A53T mice by ligating deep cervical lymph nodes. They found that reduced lymphatic influx of CSF tracers accompanied perivascular aggregation of α-syn and impaired polarization of AQ4 expression in the substantia nigra. In a study establishing an animal model of PD by administering 1-methyl-4-phenyl-1,2,3,6-tetrahydropyridine, mutant mice lacking AQP4 were shown to be significantly more susceptible to 1-methyl-4-phenyl-1,2,3,6-tetrahydropyridine (MPTP)-induced neurotoxicity than their wild-type littermates, confirming an important role for AQP4 in the process of MPTP neurotoxicity and suggesting that therapeutic strategies targeting astroglial modulation with AQP4 may offer great potential for the development of new therapies for PD (65). Therefore, meningeal lymphatic vessels have an important role in the clearance of α-syn. Using dynamic contrast-enhanced MRI to assess meningeal lymphatic flow in patients with PD, it was found that patients with PD exhibited significantly reduced meningeal lymphatic flow along with the superior sagittal and sigmoid sinuses and significantly delayed perfusion of deep cervical lymph nodes (56). Improving meningeal lymphatic drainage may be a promising therapeutic target for slowing or even preventing PD progression.

### Lymphatic Circulation and Neuroinflammatory Disorders

In CNS, in addition to neurodegenerative diseases and trauma, the development of neuroinflammatory diseases is also closely related to the drainage of meningeal lymphatic vessels. The most prevalent and widely studied disease in this category is MS, which is characterized by loss of myelination of oligodendrocytes in CNS neurons and is also thought to be caused by autoimmune destruction of these cells (66, 67). The discovery of meningeal lymphatic vessels provides an alternative route for CSF-containing macromolecules and immune cells to drain into the deep cervical lymph nodes while providing a new target for the treatment of neuroinflammatory diseases. Experimental results from MS mouse models indicated that CD4+ T cells were the main driver of the neuroinflammatory process, in addition to the genetic association of MS with MHC II haplotypes and molecules involved in the regulation of MHC II-restricted T cell-mediated inflammation supported this view (68). It has also been reported that cells of the B-lymphocyte lineage are a predominant component of immune inflammation in the brain and spinal cord of patients with MS compared to many other neuroinflammatory diseases (69). However, it is possible that in MS lesions, B cells may enhance T-cell-mediated inflammatory responses by presenting effective autoantigens (67). The meninges were mainly populated by central memory T cells (70), which accumulated in deep cervical lymph nodes by injecting *in vitro*-generated central memory CD4+ T cells into a mouse model (32).

In the past, lymphangiogenesis in a VEGFC-VEGFR3-dependent manner was observed in experimental autoimmune encephalomyelitis models, and the increased meningeal lymphangiogenesis contributes to the management of fluid accumulation and immune surveillance induced by neuroinflammation (71). Approximately 75% of patients with optic neuromyelitis optica spectrum disorder (NMOSD) have been reported to have antibodies against AQP-4 (72). AQP4 antibodies are mainly of the IgG1 isotype, and AQP4 antibodies induce IL-6 production by AQP4-expressing astrocytes, and IL-6 signaling to endothelial cells can reduce the function of the blood-brain barrier (73). By binding to the extracellular structural domain of the AQP4 receptor, AQP4 antibodies cause complement and cell-mediated astrocyte damage in addition to internalization of the glutamate transporter excitatory amino acid transporter 2 (EAAT-2), followed by granulocyte infiltration with oligodendrocyte damage and demyelination (74).

### Lymphatic Circulation and Small Vessel Diseases of CNS

Cerebral small vessel disease (CSVD), one of the leading causes of AD development and cognitive decline in the elderly, includes amyloid angiopathy, arteriolosclerosis, and other inherited small vessel diseases (75, 76). CVSD mainly affects small intracranial penetrating arteries, veins, and capillaries and can cause damage to the white matter and deep gray matter. In CSVD, the most common pathological manifestations are dilatation of the perivascular space, deposition of Aβ and other abnormal proteins (77). Moreover, dysfunction of the lymphatic system resulted in dynamics impairment of CSF/ISF, so the accumulation of abnormal proteins such as Aβ in the tissues and the subsequent dilatation of the perivascular space were correlated (78, 79). Elevated drainage of the intracranial lymphatic system was observed to reduce reactive astroglia and microglia, normalize AQP4 expression and its polarization, which reduced Aβ deposition and increased cortical and hippocampal synaptic integrity in mice (78). These provide a theoretical basis for the influence of meningeal lymphatic vessels on CSVD.

Cerebral small vessel disease is most commonly associated with arteriolosclerosis, i.e., hardening and decreased elasticity of small arteries and arterioles, and most commonly associated with hypertension, MRI was performed after intrathecal contrast injection in a hypertensive rat model with normal rats in the control group, and ventricular contrast reflux was observed only in the hypertensive group, demonstrating abnormal CSF/ISF dynamics and impaired lymphatic clearance in hypertensive rats (80). Therefore, untreated hypertension may impair the function of the lymphatic system, thus accelerating brain aging in patients with hypertension. It has been shown that cerebral amyloid angiopathy involves the same pathological features of Aβ deposition as AD, that CSF influx and ISF clearance in the brain were significantly inhibited in the relevant mouse models, and that Aβ deposition occurred before CSF/ISF dynamics were suppressed (81).

Other CSVD includes NOTCH3 mutations causing cerebral autosomal dominant arteriopathy with subcortical infarcts and leukoencephalopathy, where vascular degeneration is highly evident in the white matter region of the brain, probably due to impaired excretion of granular osmiophilic material as a result of CSF/ISF dynamic dysfunction (79, 82). And familial CSVD associated with COL4A1 may lead to hemorrhagic and ischemic strokes in adults with imaging features of microhemorrhage and leukoaraiosis (83). The pathogenesis is due to changes in the cerebrovascular basement membrane caused by COL4A1, which results in impaired CSF/ISF dynamics (84).

### Lymphatic Circulation and Stroke

The causes of stroke are diverse and the pathogenesis varies, ultimately leading to brain parenchymal necrosis, characterized by endothelial dysfunction and neuroinflammation, with the main pathological processes in the early stages of damage being resident inflammatory cells, rapid activation of inflammatory cytokines, and intercellular nuclear factors' translocation (85, 86).

In a study of MRI assessment of lymphatic vessel function after stroke, impaired ISF dynamics were observed by establishing a mouse model of stroke. However, ISF dynamics were not altered after carotid ligation or cerebral hemorrhage, and obstruction of meningeal lymphatic vessel drainage after acute subarachnoid hemorrhage exacerbated cerebral ischemia and cerebral edema (76, 87). In the acute phase after ischemic stroke and subarachnoid hemorrhage (SAH), the lymphatic system was severely impaired, but intracerebroventricular injection of tissue-type fibrinogen activator to clear the perivascular space or intravenous injection to restore arterial patency improved lymphatic system function, further confirming the association between stroke and impaired lymphatic drainage (76). Experiments confirmed that injection of fluorescent tracer into the stroke mouse model revealed a decrease in tracer drainage into the deep cervical lymph nodes and the influx into the brain parenchyma, further confirming impaired lymphatic drainage in stroke mice, and also observing the impaired function of astrocyte AQP4, leading to accumulation of Tau protein in the brain (88). Studies of cognitive impairment and pathological mechanisms associated with tauopathy in a rat model of poststroke dementia showed that stroke exacerbated cognitive dysfunction and tau hyperphosphorylation in the rat model by interfering with the clearance of tau through the lymphatic system (89). It also suggests that enhancing the drainage capacity of the meningeal lymphatic vessels and improving the clearance of brain metabolic wastes, including tau, may be an effective therapeutic approach to prevent poststroke dementia.

### Lymphatic Circulation and Glaucoma

The optic nerve is a white matter bundle of the CNS, embedded in three meningeal layers and surrounded by CSF with a peripheral pressure equal to the intracranial pressure (90). Dysfunction of the vascular system linking the retina to the optic nerve may be an important factor in retinal diseases such as glaucoma (91).

Experiments confirmed that Aβ in the vitreous and retina can be cleared through the glial water channel AQP4 pathway (76). In relevant animal experiments, intra-axial Aβ was drained into the lymphatics through the perivascular space, and Aβ efflux could be enhanced by light-induced pupillary constriction, which was attenuated by atropine or increased intracranial pressure (92). Meanwhile, the perineural space around the cranial nerves is known to provide some degree of CSF drainage to the peripheral lymphatics (93). Cheng et al. (94) have demonstrated experimentally that vagus nerve stimulation enhances the influx of CSF tracers. Furthermore, the researchers observed evidence of retrograde CSF flow into the perivascular space of the optic nerve, demonstrating that the eye-to-CSF pathway supports waste removal from the retina and vitreous (92). In addition, in humans, where ISF has been proposed to communicate between brain and eye, silicone oil has been used as an intraocular filling, and there have been some reports of silicone oil migration into the periorbital space, chambers of the eye, and cerebral ventricle (95–97). Reduced and dysfunctional lymphatic drainage increases the accumulation of macromolecular material, which can exacerbate the development of glaucoma and may be associated with pseudoexfoliative glaucoma (98). Neuroradiological examination suggested a neurodegenerative origin of glaucoma, with impaired cerebrospinal fluid dynamics and reduced clearance of Aβ amyloid due to impaired lymphatic drainage playing an important role in this process (99).

### Lymphatic Circulation and Tumor Immunology

Brain tumors have been known to elicit a potent antitumor immune response (100). More than a decade ago, Calzascia et al. (101) demonstrated through animal models that intracranial soluble tumor antigens could be drained *via* the lymphatic pathway, with tumor antigens draining through the CNS lymphatic system to the cervical lymph nodes, thereby stimulating specific immune T cells. In established mouse glioma models, function-blocking antibodies against CTLA-4 and PD-1 resulted in increased CD8+ T cells, macrophages, and natural killer (NK) cells with decreased regulatory T cells in brain tumor areas, and obtained a durable antitumor activity in mouse models (102–104). The same results were also obtained in clinical trials (103). It was demonstrated that if the cervical lymph nodes of mice were removed, an explicit reduction in immune infiltration response could occur, while anti-PD-1/CTLA-4 treatment-induced tumor regression was also eliminated (100). Current research on the relationship between meningeal lymphatic vessels and intracranial tumors has focused on glioblastoma multiforme (GBM) and intracranial melanoma. GBM is the most common and most aggressive malignant tumor of the CNS in adults with a 4–5% probability of 5-year survival rate and a 2-year survival rate of 26–33%. GBM median survival timeline is 14–15 months (105, 106). For intracranial metastatic tumors, metastatic melanoma has the highest risk of spread to the CNS among common cancers; it has a marked tendency to metastasize to the brain, characterized by ventricular and meningeal metastases (107, 108). Hu et al. (100) demonstrated that meningeal lymphatic vessels play an important role in anti-PD-1/CTLA-4 combination therapy for GBM by establishing a mouse model of GBM, and ablation of the dorsal meningeal lymphatic vessels in the tested mice significantly reduced the effect of anti-PD-1/CTLA-4 combination therapy. They also observed that in a mouse model of metastatic melanoma established with B16 cells, ablation of experimental mouse meningeal lymphatic vessels similarly reduced the efficacy of checkpoint suppression of intracranial melanoma tumors, suggesting that treatment of intracranial melanoma is partially dependent on meningeal lymphatic vessels. It has also been reported that dendritic cells can be transported from intracranial tumors to deep cervical lymph nodes *via* meningeal lymphatic tracts, and the antigen presentation involved in this process may activate CD8+ T cells and thus exert antitumor effects (109). Ablation of dorsal meningeal lymphatic vessels resulted in a significant reduction in dendritic cell drainage from deep cervical lymph nodes in mice models of GBM and melanoma (100). When model mice with GBM and melanoma overexpressed VEGF-C, the anti-PD-1/CTLA-4 combination treatment showed a good effect, which was abolished when CCL21/CCR7 blockaded, suggesting an augmented effect of VEGF-C on checkpoint treatment *via* the CCL21/CCR7 pathway (100). Furthermore, a clinical study on metastatic melanoma reported that VEGF-C concentrations positively correlated with long-term patient response to peripheral combined checkpoint blockade (110). Using a GBM mouse model (111), it demonstrated that immune responses to brain tumors can be influenced by manipulating the meningeal lymphatic vessels system and that CD8+ T cell-mediated immunity to GBM is very limited when tumors are confined to the CNS, resulting in uncontrolled tumor growth. However, ectopic expression of VEGF-C promotes enhanced initiation of CD8+ T cells in deep cervical lymph node drainage, migration of CD8+ T cells to the tumor, rapid clearance of GBM, and a durable antitumor memory response. GBM and melanoma induced remodeling of meningeal lymphatic vessels mainly through the VEGF-C/CCL21 signaling pathway, which also illustrated the role of meningeal lymphatic tracts in the tumor microenvironment of the brain tumors and provided new targets for brain tumor treatment.

## Future Perspectives on Lymphatic Circulation Research

Although meningeal lymphatic vessels no longer develop in adulthood, meningeal lymphatic vessels have plasticity (35, 36), and new therapies targeting meningeal lymphatic vessels for the above-mentioned diseases may be feasible. From the current findings, future research should be directed to include all aspects of CSF clearance, especially the portion through the meningeal lymphatic vessels. As mentioned above, AQP4 plays an important role in intracranial lymphatic drainage for the clearance of intracranial macromolecules. As a major transport channel in the CNS, AQP4 is expressed in the final foot of perivascular astrocytes and the astrocyte membrane, which faces the ependymal cells and pia mater. Intracranial AQP4 is predominantly distributed along the end-feet membranes of perivascular astrocytes. When the AQP4 gene is knocked out in normal mice, mice exhibit higher intracranial pressure, neurological deterioration, and vasogenic edema (2). Abnormal water balance is associated with a variety of neurological disorders, namely, cerebral edema, epilepsy, stroke, and tumor (84, 112, 113). It was found that AQP4 is aberrantly expressed and localized in reactive astrocytes from patients with neurodegenerative diseases. At the same time, several experiments have confirmed that AQP4 agonists can promote interstitial Aβ clearance in the brain (25, 40, 55, 114–117). AQP4 is a target antigen for autoantibody biomarkers in optic neuromyelitis optica (118). A rat model of multiple microinfarcts showed that AQP4 expression was reduced and induced lymphatic dysfunction, leading to white matter damage and cognitive impairment in experimental mice (119). The relationship between meningeal lymphatic vessels and AQP4 expression involves multiple intracranial pathological changes and holds new promise for immunotherapy of neurodegenerative diseases and tumors that deserve further exploration.

Microglia and macrophages residing in the brain showed disease-specific activation profiles in response to neurodegenerative diseases, brain injury, and brain tumor models in some cases (120). The meningeal lymphatics are closely related to the pathophysiology of the models described above, and future exploration of the relationship between altered meningeal lymphatic function and activation of abnormal intrinsic brain microglia and macrophages (5).

Treatment of aged mice with VEGF-C improved cognitive performance by allowing drainage of meningeal lymphatic vessels to deep cervical lymph nodes (5). A decrease in immune cells draining through the meningeal lymphatics may lead to the increased frequency of T cells, altered macrophage, and dendritic cell phenotypes observed in the meninges and brain of aged mice. Importantly, changes in T cell responses were also observed in human AD and PD, with increased responsiveness of peripheral helper T cells and cytotoxic T cells to α-synuclein peptides in patients with PD and clonally expanded CD4 and CD8 T cells specific for two Epstein-Barr virus antigens found in the CSF of patients with AD (121). Future experiments to assess whether the beneficial effects of VEGF-C in aged mice/patients are achieved by improving meningeal lymphatic solute drainage and modulating intracranial immune status would provide a valuable strategy for the treatment of neurodegenerative diseases.

## Summary and Conclusions

In this review, we discussed various diseases or disorders related to intracranial lymphatic drainage. Although these diseases are not classified as a whole, we believe that they all share a common underlying pathophysiological mechanism, namely, intracranial lymphatic drainage dysfunction. The intracranial lymphatic system is a bulk-flow waste removal network within the CNS, thus if damaged, it will reduce the removal of aggregated proteins such as Aβ and tau, which in turn increases the incidence of neurodegenerative diseases, among others. Although a large number of experiments have been conducted to confirm the potential of meningeal lymphatics to reduce intracranial lymphatic system-related diseases and AQP4 channels have been identified as potential drug targets, no drugs have been developed for clinical application. Future research remains focused on regulating AQP4 quantity and polarization, and how to improve lymphatic clearance.

## Author Contributions

FC and XX: conceptualization and data curation. FC and LW: funding acquisition. LW: project administration. FC: writing—original draft. All authors contributed writing—review and editing. All authors contributed to the article and approved the submitted version.

## Funding

This study was supported by the Fund of Tang Du Hospital (No. 2021YFJH005 and No. 2021SHRC033).

## Conflict of Interest

The authors declare that the research was conducted in the absence of any commercial or financial relationships that could be construed as a potential conflict of interest.

## Publisher's Note

All claims expressed in this article are solely those of the authors and do not necessarily represent those of their affiliated organizations, or those of the publisher, the editors and the reviewers. Any product that may be evaluated in this article, or claim that may be made by its manufacturer, is not guaranteed or endorsed by the publisher.
